# A vaccine targeting the L9 epitope of the malaria circumsporozoite protein confers protection from blood-stage infection in a mouse challenge model

**DOI:** 10.1038/s41541-022-00457-1

**Published:** 2022-03-08

**Authors:** Lucie Jelínková, Yevel Flores-Garcia, Sarah Shapiro, Bryce T. Roberts, Nikolai Petrovsky, Fidel Zavala, Bryce Chackerian

**Affiliations:** 1 Department of Molecular Genetics and Microbiology, University of New Mexico School of Medicine, Albuquerque, NM USA; 2 W. Harry Feinstone Department of Molecular Microbiology and Immunology Malaria Research Institute, Johns Hopkins Bloomberg School of Public Health, Baltimore, MD USA; 3 Vaxine Pty Ltd., 11 Walkley Avenue, Warradale, Adelaide 5046 Australia; 4 College of Medicine and Public Health, Flinders University, Adelaide, 5042 Australia

**Keywords:** Conjugate vaccines, Protein vaccines

## Abstract

Pre-erythrocytic malaria vaccines that induce high-titer, durable antibody responses can potentially provide protection from infection. Here, we engineered a virus-like particle (VLP)-based vaccine targeting a recently described vulnerable epitope at the N-terminus of the central repeat region of the *Plasmodium falciparum* circumsporozoite protein that is recognized by the potently inhibitory monoclonal antibody L9 and show that immunization with L9 VLPs induces strong antibody responses that provide protection from blood-stage malaria in a mouse infection model.

## Main Text

**Fig. 1:**
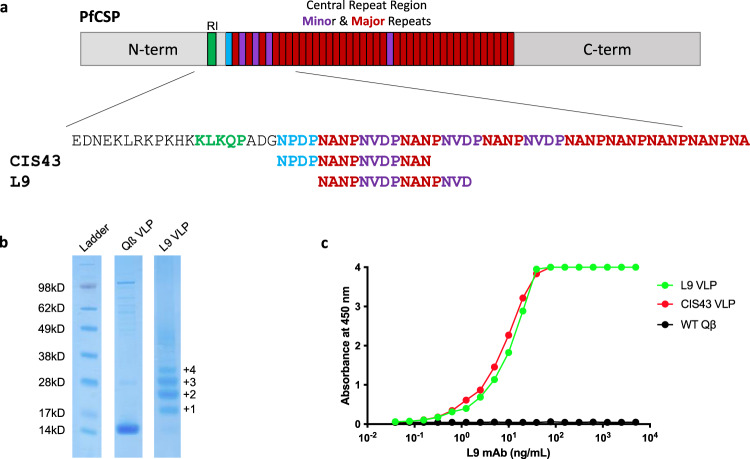
Characterization of L9 VLPs. **a** The structure of PfCSP and the location of the epitopes targeted in this study. CSP contains an N-terminal region, which contains the RI cleavage site (in green), a Junction region (between RI and the central repeat), and the central repeat (CR) Region, which contains four NVDP minor repeats (purple) and >35 NANP major repeats (red). The two peptide epitopes displayed on CIS43 and L9 VLPs are shown. **b** SDS-PAGE analysis of unconjugated (center lane) or L9 peptide conjugated (right lane) Qβ VLPs. The ladder of bands in the L9 VLP lane reflect individual copies of coat protein modified with 1, 2, 3, or more copies of the L9 peptide. Gel images are derived from the same experiment and were processed in parallel. Size markers are shown in the left lane. The unmodified gel is shown in Supplementary Fig. 1. Data from a single conjugation reaction that is representative of >6 independent reactions is depicted. **c** Binding of the L9 mAb to L9 VLPs (green), CIS43 VLPs (red), or wild-type (unmodified) Qβ VLPs (black) as measured by ELISA. This experiment was performed twice, data from one representative experiment is shown.

Pre-erythrocytic malaria vaccines (such as RTS,S^1^) that largely target the immunodominant central repeat (CR) region of the *Plasmodium falciparum* circumsporozoite protein (*Pf*CSP)—a protein that densely covers the surface of invading sporozoites—provide moderate protection from human infection^2–4^. However, the recent identification of human monoclonal antibodies (mAbs) from human volunteers immunized with an experimental irradiated whole sporozoite vaccine developed by Sanaria (*Pf*SPZ) that target epitopes in CSP outside of the CR and potently protect animal models from malaria infection have pointed to new sites of vulnerability in CSP that may be exploited using epitope-targeted vaccines^5–10^. We previously showed that a bacteriophage Qß-based virus-like particle (VLP) vaccine that multivalently displays a peptide representing the epitope of the CIS43 mAb, which is located at the junction between the N-terminal region of CSP and the CR^5^, could elicit extremely durable and high-titer anti-CSP antibody responses and reduce parasite liver burden in a mouse malaria challenge model, but did not prevent blood-stage parasitemia^10^. Here, we assessed the immunogenicity and protective efficacy of VLPs displaying the epitope of the L9 mAb, a newly described antibody that is one of the most potent anti-CSP mAbs at inhibiting parasite invasion in mouse models^6^. The L9 epitope overlaps with the CIS43 epitope, but is centered on the minor repeat sequences at the N-terminus of the CR (Fig. 1a).

**Fig. 2:**
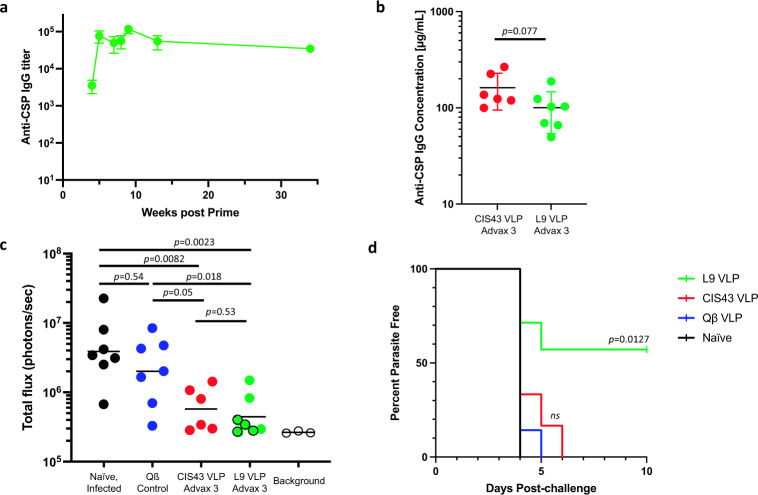
L9 VLPs elicit strong and long-lasting anti-CSP antibody responses that protect against parasitemia. **a** Mean anti-CSP IgG concentrations sampled over 34 weeks in Balb/c mice (*n* = 6/group) immunized three times (at weeks 0, 4, and 7) with L9 VLPs without adjuvant. Error bars represent SEM. **b** Anti-CSP antibody concentrations in C57BL6 mice immunized three times with CIS43 VLPs or L9 VLPs (both with Advax-3 adjuvant; *n* = 6–7/group) collected 26 days following the third immunization. Antibody concentrations were compared by two-tailed *t*-test. Serum from control (Qß VLP) immunized mice had anti-CSP antibody concentrations below the limit of detection of the assay (<0.1 ng/mL). **c** Parasite liver load (as measured by luminescence) in CIS43 VLP and L9 VLP-vaccinated (or control) C57BL6 mice (*n* = 6–7/group) following mosquito challenge. The four mice in the L9 VLP group that were protected from blood parasitemia are denoted by green circles with a black outline. A two-tailed Mann–Whitney test was used to statistically compare groups. Background luminescence was determined using three uninfected mice. **d** Percent of parasite-free mice post-challenge as evaluated by Giemsa blood smear. Log-rank test was used to statistically compare the L9 VLP and CIS43 VLP groups to the wild-type Qß VLP control group; ns not significant.

We generated Qß VLPs that multivalently display the 15 amino acid L9 epitope (L9 VLPs) by chemically conjugating a synthetic L9 peptide to the surface of VLP using a bifunctional crosslinker. L9 VLPs display an average of 385 copies of the peptide epitope per VLP (Fig. 1b), a high valency that is associated with strong immunogenicity^11^, and the L9 VLPs are strongly bound by the L9 mAb (Fig. 1c). L9 mAb also binds strongly to CIS43 VLPs, likely because of the overlap between the two peptide epitopes. L9 VLPs are highly immunogenic in mice; three doses of unadjuvanted L9 VLPs elicited high titer and durable anti-CSP antibodies (Fig. 2a), as was previously observed in mice immunized with CIS43 VLPs^10^. Similar to CIS43 VLPs, L9 VLPs elicit antibodies that inhibit binding of both the L9 mAb and the CIS43 mAb to CSP (Supplementary Fig. 2), indicating that induced antibodies bind to the junctional region of CSP.

To test whether L9 VLPs could confer protection from malaria challenge, C57Bl/6 mice were vaccinated with L9 VLPs, CIS43 VLPs, or, as a negative control, wild-type Qß VLPs and then challenged with malaria-infected mosquitoes. We have previously shown that co-administration of CIS43 VLPs with the adjuvant Advax-3, which is a mixture of CpG55.2 oligonucleotide (a TLR9 agonist) with aluminum hydroxide, could increase anti-CSP antibody levels, so all vaccines were adjuvanted with Advax-3. As is shown in Fig. 2b, both L9 VLPs and CIS43 VLPs mixed with Advax-3 elicited strong anti-CSP antibody responses. After three immunizations, mice were exposed to mosquitoes infected with luciferase-reporter containing transgenic *P. berghei* (*Pb*) engineered to express full-length *Pf*CSP in place of *Pb*CSP (*Pb-Pf*CSP-*Luc*)^12^. Forty-two hours after challenge, liver parasite loads were measured using an intravital imaging system. Mice immunized with CIS43 VLPs and L9 VLPs had significantly lower liver parasite loads than control VLP-vaccinated mice or unvaccinated (naïve) controls (Fig. 2c). Relative to naïve mice, immunization with CIS43 VLPs reduced mean parasite loads by ~92% (similar to what we showed previously^10^) and immunization with L9 VLPs reduced mean parasite loads by ~95%. Mice in the L9 VLP vaccinated group had slightly lower mean liver parasite loads than mice in the CIS43 VLP group (~35% lower, after subtracting background luminescence), but this difference was not statistically significant (*p* = 0.53). Beginning four days after infection, blood smears from mice were evaluated for parasitemia. While all control mice and CIS43 VLP-immunized mice developed blood-stage parasitemia, four of the seven mice immunized with L9 VLPs remained parasite-free 10 days after infection, indicating that these mice were protected from blood-stage infection (Fig. 2d).

Here, we evaluated and compared the efficacy of vaccines targeting two overlapping epitopes within the junctional/minor repeat regions of *Plasmodium falciparum* CSP that are recognized by the potent inhibitory monoclonal antibodies, CIS43 and L9. VLPs displaying both epitopes could elicit strong anti-CSP antibody responses and could significantly reduce parasite liver loads in experimentally challenged mice. However, even a single infected liver cell can seed the blood stage of the parasite lifecycle. Because the luminescence assay used to detect liver infection is not sensitive enough to predict sterilizing protection, we also evaluated blood smears in vaccinated mice and showed that only immunization with L9 VLPs could prevent blood parasitemia in a subset of mice. The 15 amino acid CIS43 and L9 epitopes overlap by 11 amino acids, suggesting that subtle changes in the epitope targeted by anti-CSP antibodies can dramatically affect protective efficacy. It has been hypothesized that the most potent anti-CSP mAbs recognize epitopes derived from the joining of minor and major tetrapeptide repeats, including DPNA (minor/major) and NPNV (major/minor)^6,13,14^. The DPNA motif is found twice in the CIS43 epitope and once in the L9 epitope, whereas NPNV is found twice in the L9 epitope and once in the CIS43 epitope. In addition, the minimal L9 binding peptide (NANPNVDP) is centered on the NPNV sequence^15^. To examine the specificity of antibodies induced by the L9 and CIS43 VLP vaccines, the binding of sera to peptides representing the L9 and CIS43 epitopes, as well as peptides representing the major repeat (NANP) and non-naturally occurring peptides representing repeat junctions (DPNA and NPNV) was assessed (Supplementary Fig. 3). Both vaccines induced antibodies that bound to all five peptides, but sera from mice immunized with L9 VLPs had stronger relative binding to the L9 epitope than the CIS43 epitope, and reacted more strongly with the (NPNV)_3_ peptide than the (DPNA)_3_ peptide. In contrast, sera from CIS43-immunized mice bound more strongly to the CIS43 epitope and the (DPNA)_3_ peptide. Thus, it is possible that antibodies which more strongly target NPNV are more functionally active and, therefore, may aid in preventing blood parasitemia. Future experiments may reveal the specific nature of vulnerability of this region of CSP. Taken together, these studies indicate that L9 VLPs are a promising malaria vaccine candidate.

## Methods

### Ethics

All animal research complied with the Institutional Animal Care and Use Committee of the University of New Mexico School of Medicine (Approved protocol #: 19-200870-HSC), Johns Hopkins University (Approved protocol permit #: MO18H419).

### Production and characterization of VLP-based vaccines

L9 VLPs and CIS43 VLPs were produced similarly; the fifteen amino acid L9 and CIS43 epitope peptides were synthesized (GenScript) and modified to contain a C-terminal *gly-gly-gly-cys* linker sequences (L9; NANPNVDPNANPNVD*GGGC*, CIS43; NPDPNANPNVDPNAN*GGGC*) and were conjugated directly to surface lysines on Qβ bacteriophage VLPs using the bidirectional crosslinker succinimidyl 6-[(beta-maleimidopropionamido) hexanoate] (SMPH; Thermo Fisher Scientific). The amine-reactive arm of SMPH was linked to surface-exposed lysines on Qβ VLPs by reacting the VLPs with SMPH at a 1:10 molar ratio. Qβ VLP-SMPH conjugates were purified by centrifugation using an Amicon Ultra-4 centrifugal filtration device (EMD Millipore). Qβ VLP-SMPH was linked to the L9 or CIS43 peptide by virtue of the exposed sulfhydryl residues on the C-terminal cysteine residue of the peptides. Qβ VLP-SMPH was reacted with the peptides at a 1:10 molar ratio and peptide conjugated VLPs were purified by centrifugation using the Amicon unit as described above. The efficiency of conjugation was assessed by gel electrophoresis using a 10% SDS denaturing polyacrylamide gel followed by analysis using ImageJ software to calculate the average peptide density per VLP. Conjugation of VLPs with the L9 peptide was performed multiple times, with similar conjugation efficiencies. Presence of the L9 peptide on L9 VLPs was confirmed by ELISA. Two hundred and fifty nanograms of VLPs were used to coat wells of an ELISA plate. Wells were probed with dilutions of mAb L9 (generously provided by Robert Seder, NIH Vaccine Research Center), followed by a 1:4000 dilution of horseradish peroxidase (HRP) labeled goat anti-human IgG (Jackson Immunoresearch). The reaction was developed using 3,3′,5,5′-tetramethylbenzidine (TMB) substrate (Thermo Fisher Scientific) and stopped using 1% HCl. Reactivity was determined by measuring optical density at 450 nm (OD_450_) using an AccuSkan plate reader (Fisher Scientific).

### Mouse immunization studies

For the initial evaluation of immunogenicity, groups (*n* = 6) of 4–5-week-old female Balb/c mice (obtained from the Jackson Laboratory) were immunized intramuscularly with 5 μg of L9 VLPs without exogenous adjuvant. Mice were boosted twice at 4 and 7 weeks after the initial prime. Challenge studies were performed using 7–8-week–old C57Bl/6 mice (*n* = 6–7/group), which are more susceptible to malaria challenge than Balb/c mice. Mice were immunized with 5 µg doses of L9 VLPs, CIS43 VLPs, or wild-type control Qß VLPs in combination with 20 µL of Advax-3 adjuvant (5 mg/mL). Mice were immunized at days 0, 28, and 56, serum was collected at day 82, and the mice were challenged at day 84. An additional group of naïve (unimmunized) mice were included in this experiment.

### Quantitating antibody responses

Serum antibodies against full-length CSP were detected by ELISA using recombinant CSP expressed in *Pseudomonas fluorescens*^16^ (and generously provided by Gabriel Gutierrez at Leidos, Inc.) as the coating antigen. Peptide ELISAs were performed using peptides representing the L9 epitope (see above), the CIS43 epitope (NPDPNANPNVDPNAN), the CR major repeat (NANPNANPNANPNANPNA), and multimeric NPNV [(NPNV)_3_; NPNVNPNVNPNV] and DPNA [(DPNA)_3_; DPNADPNADPNA) peptides. All peptides were synthesized to contain a C-terminal -*GGGC* linker sequence. For CSP ELISAs, Immulon 2 plates (Thermo Scientific) were coated with 250 ng of CSP in 50 μL PBS and incubated at 4 °C overnight. For peptide ELISAs, Immulon 2 plates were coated with 500 ng streptavidin (Invitrogen) for 2 h at 37 °C. Following washing, SMPH was added to wells at 1 μg/well and incubated for 1 h at room temperature. Specific peptides were added to the wells at 1 μg/well and incubated overnight at 4 °C. For all ELISAs, wells were blocked with PBS-0.5% milk for 2 h at room temperature. Sera isolated from immunized animals were serially diluted in PBS-0.5% milk and applied to wells and incubated at room temperature for 2.5 h. Reactivity to the target antigen was detected using HRP-labeled goat anti-mouse IgG (Jackson Immunoresearch, diluted 1:4000). Reactivity was determined using TMB substrate. End-point dilution titer was defined as the greatest sera dilution that yielded an OD_450_ > 2-fold over background. For mouse sera, anti-CSP antibody concentrations were also quantitated by ELISA by generating a standard curve using known concentrations of the anti-CSP mouse mAb 2A10^7^. Anti-CSP antibody concentrations were determined by generating a standard curve using known concentrations of the anti-CSP mouse mAb 2A10. Competition ELISAs were performed by using the CSP ELISA protocol with the following modifications: after serum from L9 VLP, CIS43 VLP, or wild-type control Qß VLP immunized mice was added to the plate, 40 ng of the human mAb L9 or CIS43 (at a final concentration of 400 ng/mL) was added to each well and incubated for 30 min. L9 or CIS43 mAb binding to CSP was detected using HRP-labeled goat anti-human IgG at a 1:4000 dilution.

### Mouse Pb-PfCSP-Luc sporozoite mosquito challenge

Mice were challenged directly by using infected mosquitos 4 weeks following their third final vaccination. *Anopheles stephensi* mosquitos were infected by blood-feeding on *Pb-Pf*CSP*-Luc* infected mice. Prior to challenge, mice were anesthetized with 2% Avertin, and then exposed to six mosquitos for a blood meal for 10 min. Following feeding, the number of mosquitos positive for a blood meal was determined. Liver luminescence was assessed 42 h post-challenge by intraperitoneally injecting anesthetized mice with 100 µL d-luciferin (30 mg/mL) and then determining liver luminescence using an IVIS Spectrum Imaging System (Perkin Elmer). Beginning 4 days after challenge, blood smears were collected daily and then evaluated by Giemsa staining for parasitemia.

### Statistics

All statistical analyses of data were performed using GraphPad Prism 9 using two-sided tests. For percent inhibition calculations (using the data shown in Fig. 2c), the mean liver luminescence values of groups of vaccinated mice was divided by the mean of mice in the naïve, infected control group. The mean background luminescence level was subtracted from all values.

### Reporting summary

Further information on research design is available in the Nature Research Reporting Summary linked to this article.

## Supplementary information

Supporting Material

Reporting Summary

## Data Availability

The datasets used and/or analyzed in the current study are available from the corresponding author upon reasonable request.
