# Supplementary material for: A vaccine targeting the L9 epitope of the malaria circumsporozoite protein confers protection from blood-stage infection in a mouse challenge model
Source: NPJ Vaccines. 2022 Mar 8;7:34. doi: 10.1038/s41541-022-00457-1 (PMC8904524; doi:10.1038/s41541-022-00457-1)
Supplement: Supplementary file 1 — Supporting Material [file 41541_2022_457_MOESM1_ESM.pdf]

Supporting information:

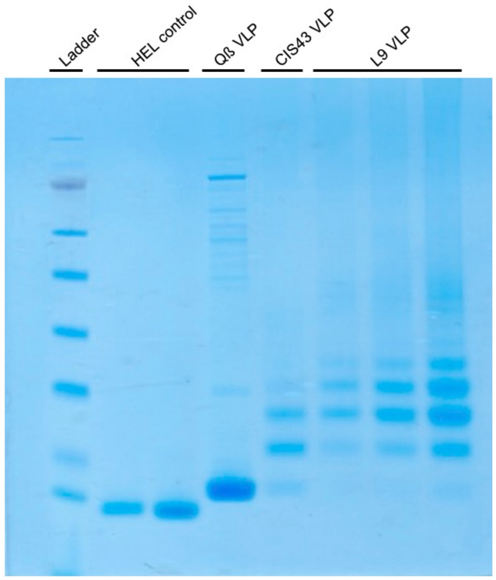

Supplementary Figure 1. Unmodified gel used as a source of the data shown in Fig. 1b. Lane 1 (left) contains molecular weight markers (Invitrogen SeeBlue Plus2 Prestained Markers). Lanes 2 & 3 contain different amounts of Hen Egg Lysozyme (Gold Biotechnology). Lane 4 contains unconjugated Qß VLPs. Lane 5 contains CIS43 peptide conjugated VLPs. Lanes 6-8 contain different amounts of L9 peptide conjugated VLPs. Lanes 1, 4, and 8 are shown in Figure 1b.

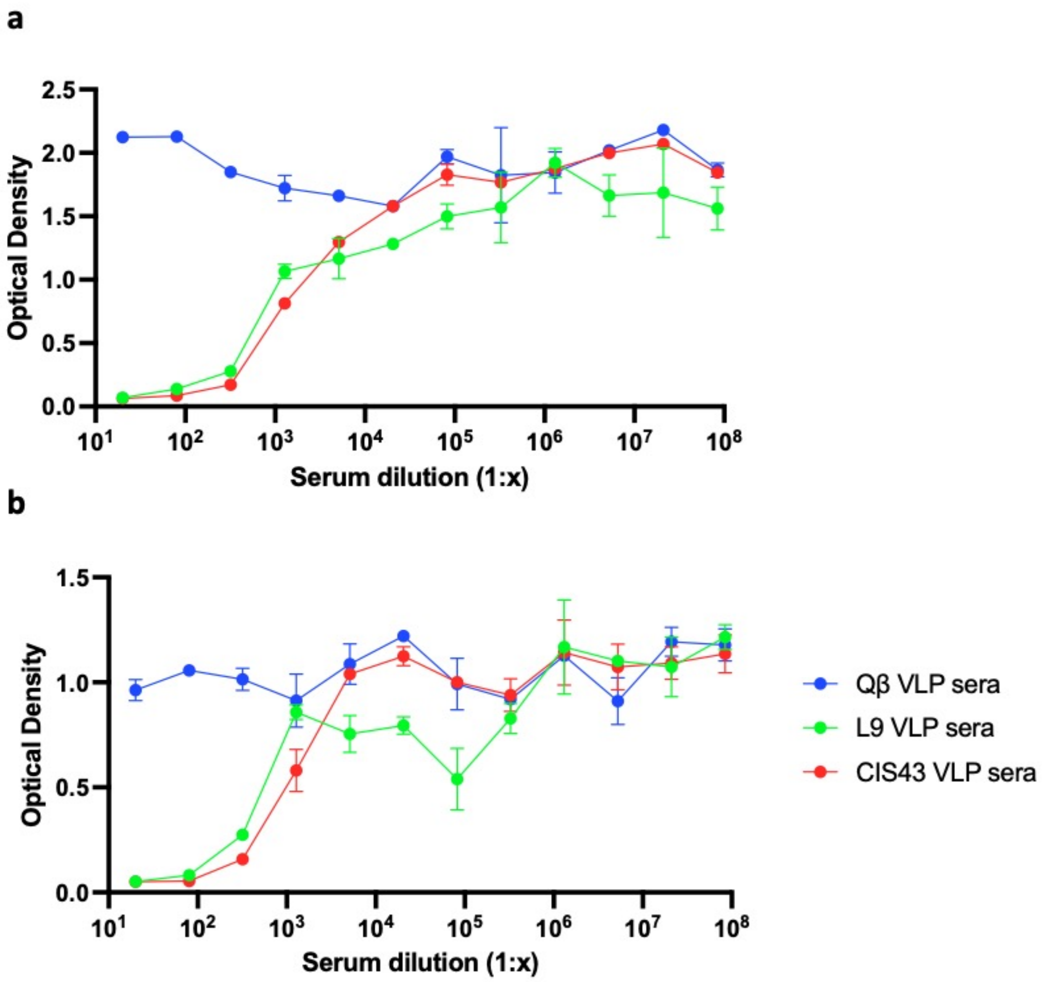

Supplementary Figure 2. Sera from mice immunized with L9 VLPs and CIS43 VLPs inhibit the binding of mAbs L9 and CIS43 to CSP. Pooled sera from L9 VLP-immunized mice, CIS43 VLP-immunized mice, or Qß VLP-immunized mice were tested by competition ELISA for inhibition of **a** L9 mAb binding to CSP, or **b** CIS43 mAb binding to CSP. This experiment was performed in duplicate, error bars represent SEM.

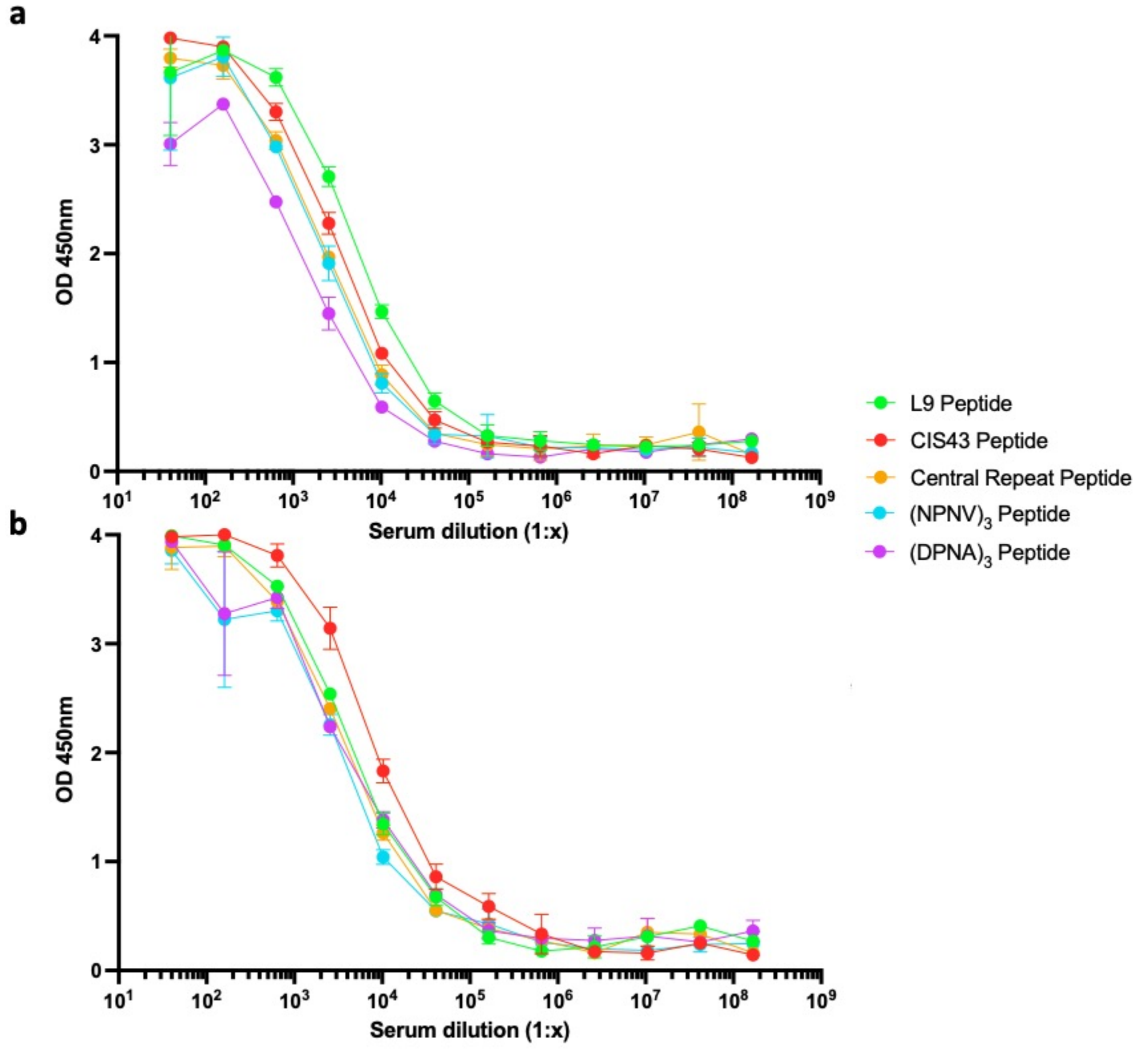

Supplementary Figure 3. Binding of sera to specific CSP-derived epitopes. Pooled sera from **a** L9 VLP-immunized mice or, **b** CIS43 VLP-immunized mice were tested for binding to five different peptides by ELISA. This experiment was performed in triplicate, error bars represent SD.
